# Assessment of a modification of Brückner’s test as a screening modality for anisometropia and strabismus

**DOI:** 10.4103/0974-620X.71890

**Published:** 2010

**Authors:** Abadan Khan Amitava, D. Kewlani, Z. Khan, A. Razzak

**Affiliations:** Department of Ophthalmology, JN Medical College, Aligarh Muslim University, Aligarh - 202 001, India; 1 Department of Community Medicine, JN Medical College, Aligarh Muslim University, Aligarh - 202 001, India

**Keywords:** Amblyopia, diagnostic test, likelihood ratio, modified Brückner test, screening test

## Abstract

**Background:**

Current amblyopia screening methods are not cost effective.

**Aim:**

To evaluate the diagnostic capability of a modified Brückner test (MBT) for amblyopiogenic risk factors.

**Materials and Methods:**

We applied the MBT using the streak retinoscope to identify anisometropia and strabismus by noting an inter-ocular difference in movement and glow, from children who failed 6/9 Snellen on community vision screening, followed by comprehensive eye examination.

**Statisitics:**

Data were analyzed by 2 × 2 tables for diagnostic test parameters (95% CI).

**Results:**

From 7998 children vision-screened, 392 failed 6/9 VA and were referred. Since 34 failed to reach the centers, and 15 were excluded due to poor/ no glow, data from 343 was analyzed. The prevalence of anisometropia of 0.5D was 17%, of 1D was 11% and of strabismus 5%. For the MBT the accuracy was ≥ 90% (95%CI 89% to 97%) over the three outcomes. The sensitivity, specificity, NPV and +LR for anisometropia of 0.5D were: 0.57 (0.48, 0.64), 0.97 (0.95, 0.98), 0.92 (0.90, 0.93) and 18 (9.7, 35); for 1D: 0.74(0.60, 0.82), 0.95 (0.94, 0.97), 0.97 (0.95, 0.98) and 16 (9.3, 28); and for strabismus: 0.5 (0.32, 0.66), 0.98 (0.97, 0.98), 0.97 (0.96, 0.98) and 20 (9.1, 42).

**Conclusion:**

Our data suggests that the MBT is highly accurate and useful for *ruling in* anisometropia and strabismus in children who fail 6/9 Snellen. The MBT needs further validation, both by different care givers and on differing populations. It offers an affordable, portable, and clinically useful tool to detect anisometropia and strabismus. We suggest that performing an MBT prior to uniocular retinosocpy should be a routine practice.

## Introduction

As recently highlighted in a 2006 workshop in Delhi, there is scarce data on refractive errors particularly in the teenage years,[1] and their impact on visual disability, including amblyopia. Anisometropia,[2] high isoametropia and strabismus remain as important amblyogenic causes. Amblyopia accounts for more unilateral blindness than all other causes combined,[3] and has a cumulative devastating effect on account of its persistence into adulthood. Depending on the population studied, and definition, the reported prevalence of amblyopia varies from 0.4% to 5.2%.[4,5] It is imperative that a successful screening strategy be available, which can be applicable in childhood, when amblyopia is most amenable to therapy. A recent careful systematic review opined that no current strategy for vision screening in childhood is cost effective.[6] Available screening strategies suffer from being either too costly, such as the photo screeners or too exhaustive (manual refraction).[7,8] One relatively simple option involves the Bruckner test, performed by using the direct ophthalmoscope to obtain a red reflex simultaneously in both eyes, to detect strabismus and moderate to severe anisometropia. We believe that the retinoscopic reflex by its very nature should be able to detect anisometropia of smaller amounts, apart from strabismus. In addition, its availability with optometrists/ and ophthalmic assistants can provide a screening tool in peripheral health set ups.

Our department of community medicine proposed to conduct a rapid epidemiological assessment of community-based visual acuity screening (employing teacher volunteers) in a district, as part of a Government of India survey. We used this opportunity to apply the modified Brückner test to those children who were referred on account of having failed the 6/9 letter test on community screening.

The specific aim of this prospective study has been to validate (adhering to the standard of reporting diagnostic accuracy protocol)[9] a modified form of Brückner test, utilizing the streak retinoscope as a screening tool to rapidly detect the presence of anisometropia and strabismus, the two main causes of amblyopia, in children who fail to reach the 6/9 line on the Snellen visual acuity (VA) chart.

## Materials and Methods

This was a prospective, diagnostic accuracy study to evaluate a modified form of Brückner test as a screening modality to identify the two most common amblyopiogenic risk factors: anisometropia and strabismus. The population consisted of school going children of 10-14 years old, from a community based, multiple cluster-sampling design. We randomly identified 40 clusters in our district: 36 rural and 4 urban, 50% from villages/ urban posts, having a junior high school, and 50% from areas which either had no school or only primary schools. In two interactive workshops teacher-volunteers were trained in VA assessment using isolated illiterate E, of size 6/9 Snellen. Four Es differently oriented were printed on a square cardboard sheet, which could be rotated to vary the presentation. A 6-m knotted rope assisted in measuring the appropriate distance. Each volunteer was assigned a minimum of 200 children; vision was checked with spectacles if they had been previously prescribed. Absent children were covered on another day; if the numbers were short, adjacent school children or neighborhood non-school going children were recruited. All children failing the 6/9 Snellen were to be referred to pre-designated local centers on predetermined dates, and would be recruited in the diagnostic accuracy study.

**Figure 1:**
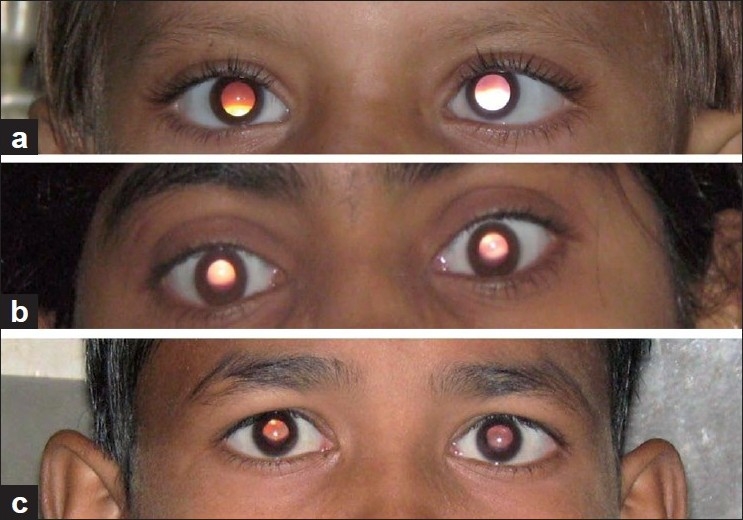
(a) Photographic Bruckner reflex in a 10 years female: Note the obvious asymmetry. Refractive data: RE: -1.0DSx-2.5DC *@* 180°, LE: -0.75DSx-1DC*@* 180°.Spherical equivalent: RE: -2.25 D, LE: -1.25 D (b) Photographic Bruckner reflex in a 14 years female. The asymmetry is noticeable. Refractive data: RE: +3DS x+2.5 DC*@* 180, LE: +3 DS × +3.5 DC *@* 180. Spherical equivalent: RE: +4.25 D, LE: +4.75 D (c) Bruckner reflex in a 13 year male. The asymmetry is apparent. Refractive data: -11 DS, and LE: -13 DS

The referral centers were manned by two experienced and trained optometrists and an ophthalmologist, with special interest in pediatric ophthalmology, and 10 years teaching experience in a medical college. Initially the modified Brückner test (the index test) was administered to all the referred children in an unlit dark room, using a streak retinoscope (Heine Beta 200 Skiaskop, Herrsching, Germany) from a working distance of 2/3 m, by a third year ophthalmology resident trained in its use in an amblyopia clinic setting. The streak was swept vertically and horizontally across both eyes. By its very nature, the vertical sweep demanded that the streak (intercept) be horizontal in which case it would cover both the eyes at the same time. Any asymmetry of the reflex both in speed and glow, between the two eyes was recorded as anisometropia [Figure 1a–c]. In addition, a difference in brightness alone was considered as indicative of lack of alignment and the child was labeled to have a strabismus. Accommodation was assumed to be stimulated only to a small, predictive amount as considered for Mohindra technique of non-cycloplegic retinoscopy at near.[10] This test was chosen since it is an objective test and can be administered to infants and preschoolers, an age most amenable to reap the benefits of anti-amblyopic therapy. Moreover it is cheap, portable and can be easily mastered. All children then underwent a detailed evaluation on the same day. We excluded children who had nystagmus or lacked a glow in one or both eyes. The comprehensive ocular examination consisted of Snellen visual acuity, Hirschberg testing, cover tests, ocular motility evaluation, stereopsis testing (on Titmus Fly), Ishihara test, dry and wet (1% cyclopentolate) retinoscopy, direct ophthalmoscopy, fixation behavior, and a magnified loupe examination of the anterior segment. The ophthalmologist and optometrists were masked to the results of the modified Brückner test. Anisometropia was considered as an inter-ocular difference in spherical equivalent on retinoscopy. We planned to evaluate the modified Brückner test at anisometropia of 0.5 and 1.0 D. Manifest strabismus was deemed to be present if the child demonstrated a decentred corneal reflex, movement on cover test, and lacked stereopsis. All children needing a refractive correction were prescribed and provided free glasses. Those needing anti-amblyopia therapy, strabismus surgery, or retinal or oculoplastic consult were advised to reach the parent facility and offered privileged care.

All details were diligently collected and regularly updated onto a computer for rapid analysis. The entire exercise needed to cover 40 clusters, with 200 children in each cluster, was planned to finally screen 8000 children and be conducted over six weeks.

Statistical analysis included estimation (with 95% confidence interval) of the prevalence of amblyopia, strabismus and anisometropia and the analysis of the 2 × 2 contingency table, using sensitivity, specificity, predictive values, likelihood ratios, diagnostic accuracy, pretest and post test probability of disease utilizing online diagnostic test and 2 × 2 contingency table calculators.[11,12] We did not undertake any test of reproducibility.

## Results

**Figure 2:**
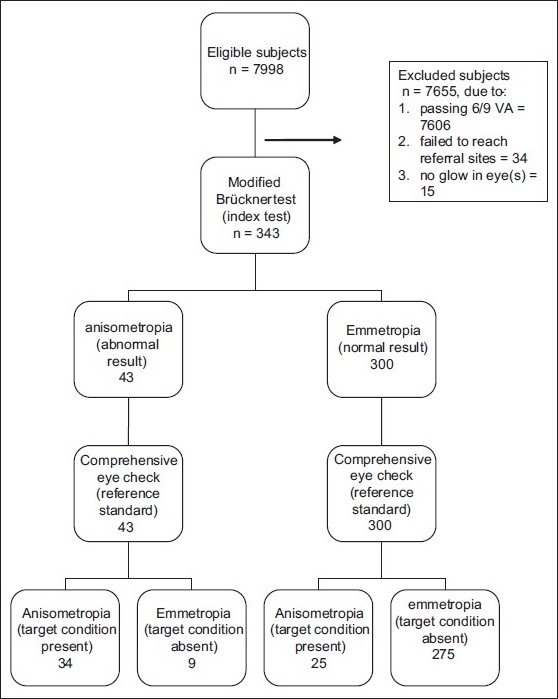
Flow diagram of the modified Brückner test when applied to detect anisometropia of ± 0.5 D

A total of 40 teacher-volunteers attended the two workshops. A total of 7998 children of 10-14 years, 55.4% of whom were males, were covered, from 39 clusters, due in part to one volunteer dropping out, and the others screening 180 to 230 students each Figure 2. A total of 392 (4.9% of the population) children were referred, on specified dates, to seven referral centers located within walking distances of the clusters. Thirty four of the 392 (8.7%) referred failed to reach the referral sites. A total of 10 visits were made to these referral centers. Of the 358 children who reached, 343 could be screened by the modified Brückner method since 15 children were excluded: cataracts (3), pseudophakia with opaque media (2), corneal opacity/ phthisis (6), and healed uveitis, empty socket, keratoconus (one each). The clinical and demographic profile of the 343 children is shown in Table 1.

**Table 1:** Clinical and demographic profile of the study population (*n*= 343) screened with modified Brückner test

*Feature*	*RE (where appropriate)*	*LE (where appropriate)*
Gender	Males: 172 (50.1%), Females: 171 (49.9%)	
School goers	320 (93.3%)	
Spectacle use	Never used: 334 (97.4%) Had worn or used: 1 (0.3%) Presently wore: 8 (2.3%)	
Visual impairment* at presentation	6/6 to 6/18: 286 (83.4%)<6/18 to 6/60: 28 (8.2%)< 6/60: 29 (8.5%)	6/6 to 6/18: 289 (84.3%)<6/18 to 6/60: 36 (10.5%)< 6/60: 18 (5.2%)
Spherical equivalent: µ±SD (range)	-0.23 ± 2.35 D (-20 D to + 8 D)	-0.18 ± 2.0 D (-17 to + 6 D)
Visual impairment* assessed after BCVA†	6/6 to 6/18: 318 (92.7%) <6/18 to 6/60: 16 (4.7%)< 6/60: 9 (2.8%)	6/6 to 6/18: 329(95.9%)<6/18 to 6/60: 10(2.9%)< 6/60: 4(1.2%)
Anisometropia (D) µ±SD (median and range)	5.0×10^-2^ D ± 1.40 D (0.0 D, -6.25 to + 20 D)	
Provided glasses	121 (35.3 %)	
Referred for privileged care	57 (16.7%)	
Referred for strabismus/amblyopia treatment	39 (11.4%)	

* Adapted and modified from International Statistical Classification of Diseases and Related Health Problems, Xth revision. Geneva, WHO, 1992

† BCVA: best corrected visual acuity, i.e. VA after refractive correction

The response of the modified Brückner test for the presence of different levels of anisometropia and strabismus is depicted in Table 2. We assessed sensitivity, specificity, likelihood ratios (LR), both positive (+LR) and negative (-LR), along with their 95% CI. We also evaluated the pre-test and post-test probability and odds ratio (OR), and the diagnostic accuracy: the last as a measure of overall correct fraction. The details are depicted in Table 3.

**Table 2:** Modified Brückner test result in detecting different levels of anisometropia and strabismus (*n* = 343)

*Anisometropia ± 0.5 D*	*Present (59)*	*Absent (284)*	*Total*
Brückner test +	34	9	43
Brückner test –	25	275	300
*Anisometropia ± 1.0 D*	*Present (39)*	*Absent (304)*	

Brückner test +	29	14	43
Brückner test –	10	290	300
*Strabismus*	*Present (18)*	*Absent (325)*	

Brückner +	9	8	17
Brückner -	9	317	326

**Table 3:** Diagnostic test parameters for the Modified Brückner test

*Test parameters (95%CI)*	*Anisometropia 0.5D*	*Anisometropia 1 D*	*Strabismus*
Prevalence*	0.17 (0.13, 0.21)	0.11 (0.08, 0.15)	0.052 (0.03 to 0.08)
Sensitivity	0.57 (0.49, 0.64)	0.74 (0.60, 0.82))	0.50 (0.32, 0.66)
Specificity	0.97 (0.95, 0.98)	0.95 (0.94, 0.97)	0.98 (0.97, 0.98)
Positive predictive value	0.79 (0.67, 0.88)	0.67 (0.56, 0.76)	0.53 (0.33, 0.70)
Negative predictive value	0.92 (0.90, 0.93)	0.97 (0.95, 0.98)	0.97 (0.96, 0.98)
Accuracy	0.90 (0.87, 0.92)	0.93 (0.90, 0.95)	0.95 (0.93, 0.97)
Prior prob (odds)	17% (0.2)	11% (0.1)	5% (0.1)
+Likelihood ratio (+LR)	18 (9.7, 35)	16 (9.3 – 28)	20 (9.1, 42.4)
Posterior prob (odd) of a positive test	79% (3.7) (66% - 88%)	67% (2.1) (55% - 78%)	52% (1.1) (32%, 81%)
-Likelihood ratio (-LR)	0.45 (0.38, 0.55)	0.27 (0.17 – 0.48)	0.51 (0.34, 0.71)
Post test prob (odds) of a negative test	9% (0.1) (6% to 11%)	3% (0.0) (2% - 6%)	3% (0.0) (2%, 4%)

* Prevalence refers to the proportion with the level of anisometropia or strabismus in the 343 children seen in the referral sites

## Discussion

Our analyses suggest that the modified Brückner test with the streak retinoscope is highly accurate (accuracy ≥90%) and performs well for *ruling in* anisometropia and strabismus in children who fail 6/9 Snellen VA. This is evident from the high +LRs: 18 (95%CI 9.7, 35) for anisometropia ≤ ± 0.5 D and 20 (95%CI 9.1, 42.4) for strabismus. This results in large increases in post test probability of diagnoses given a positive test result. The specificity and LR change little even when we evaluate the test on children of refractive error ≤ ± 4 D, suggesting that the test performs well in a population with low ametropia. Vision screenings undertaken by teachers have been successfully carried out in India.[13] The application of the modified Brückner test to such a screened population would be a practical application of such a test.

Kothari has already highlighted the importance of the standard Brückner test in detecting significant refractive errors in children,[14] although we disagree with the use of the term ‘screening test’ since it is applied to a population in a clinic setting. The term ‘case detection test’ may be more appropriate in this setting.[15] This study reported the prevalence of ametropia as 63.5%. This is quite contrary to the prevalence of ametropia in the community, which is known to be around 2.6% to 4.9%.[16,17] Importantly data collected and analyzed while quoting only sensitivity/ specificity and positive and negative predictive values are only of limited value. This is because the former are population measures which look ‘backward’, while predictive values are affected by the prevalence of the disease.[18] More pertinent measures, both forward looking and applicable to varying prevalence situations, are the LRs.[19] Reanalysis of Kothari’s data yields a +LR of 3.4 (95%CI 2.3, 5) and a –LR of 0.11 (95%CI 0.06, 0.21): suggesting that the test is not very good to rule in ametropia, but more useful to identify the emmetrope. This would limit its use in a screening environment.

Photographic Brückner test has been recommended as a screening modality since 1994, when a study noted its high sensitivity (82%), specificity (91%) and accuracy (84%).[20] This calculates to a high +LR of 9.1, and a low –LR of 0.2: both suggesting that it is a good screening modality. The medical technology innovations (MTI) photoscreener performed significantly better among pediatric residents, when compared to the Brückner reflex in detecting reflex asymmetry.[21] Both these options are too costly and have not found universal applicability in developing countries.

Interestingly in the UK there is a raging debate about the benefits of (and the appropriate age for) screening for visual disorders, with no formal screening for amblyopia being recommended in preschool years.[22,23] There exists evidence that screened infants have a significantly lower prevalence and severity of amblyopia when compared to non-screened groups.[24,25] Most benefit-to-cost analyses reveals that equipment based screening appears prohibitively costly;[26] although the recent success of the now inexpensive and commonly available digital cameras as a means of successful screening utilizing the enhanced Brückner reflex needs to be validated in India.[27]

We confess that our study merely serves to suggest that this novel use of the retinoscope to screen for amblyopiogenic risk factors should be seriously considered. Its drawbacks are that it was not carried out on appropriate age groups and was applied as a second stage tool in children who failed 6/9 Snellen. Nevertheless, we believe that the modified Brückner test needs to be further evaluated, both by optometrist and ophthalmic assistants as also pediatricians, and in younger preschool children, as also in special groups such as school dropouts. Studies are needed where the MBT can be compared to the usual Bruckner test in population-based screening scenarios. At the present, it offers a relatively low cost and clinically useful method of detecting anisometropia and strabismus, both significant disorders impacting vision at an age when intervention can still be of great benefit. Perhaps it should be regularly attempted prior to performing uniocular retinoscopy.
